# Designing all-pay auctions using deep learning and multi-agent simulation

**DOI:** 10.1038/s41598-022-20234-3

**Published:** 2022-10-08

**Authors:** Ian Gemp, Thomas Anthony, Janos Kramar, Tom Eccles, Andrea Tacchetti, Yoram Bachrach

**Affiliations:** DeepMind, London, UK

**Keywords:** Computer science, Applied mathematics

## Abstract

We propose a multi-agent learning approach for designing crowdsourcing contests and All-Pay auctions. Prizes in contests incentivise contestants to expend effort on their entries, with different prize allocations resulting in different incentives and bidding behaviors. In contrast to auctions designed manually by economists, our method searches the possible design space using a simulation of the multi-agent learning process, and can thus handle settings where a game-theoretic equilibrium analysis is not tractable. Our method simulates agent learning in contests and evaluates the utility of the resulting outcome for the auctioneer. Given a large contest design space, we assess through simulation many possible contest designs within the space, and fit a neural network to predict outcomes for previously untested contest designs. Finally, we apply mirror ascent to optimize the design so as to achieve more desirable outcomes. Our empirical analysis shows our approach closely matches the optimal outcomes in settings where the equilibrium is known, and can produce high quality designs in settings where the equilibrium strategies are not solvable analytically.

## Introduction

Many economic allocation decisions are determined by a competition for a prize based on expending costly efforts. For example, multiple political candidates may engage in costly political campaigns, but only one candidate wins; though only the winner is rewarded, other candidates cannot recover their expenditure. Similarly, Netflix offered a prize of one million dollars in an open competition to improve its recommender system^1^. Again, only the winning entry gets the prize, but other participants incur the cost of their effort.

Such contests are modelled in the economic literature as *All-Pay auctions*^2–5^, where players simultaneously bid for a fixed prize; the highest bidder receives the prize, and *every* player, including non-winners, pays their bid. A key question regarding All-Pay auctions is how to design them to optimize the utility achieved by the auctioneer. For instance, should the auctioneer give all the reward to the top entry, or does it make sense to give some of the reward to the top entry, and some to the second entry?

Earlier research has investigated how different auction designs affect the utility of the auctioneer^5–8^. Such work examines a specific model of the All-Pay auction given as a normal-form game and analytically solves for the Nash equilibrium of the bidding strategy, expressed as a probability distribution over the possible bids. This approach has multiple limitations. First, economists have only managed to solve for the Nash equilibrium under very specific auction designs. Secondly, in many settings, participants are likely to adjust their bidding strategy by using simple learning behaviors based on their experience^9–11^, so one cannot always assume the Nash equilibrium behaviour as a model of participants’ behavior when designing the auction.

*Our Contribution*: We propose a *machine learning method for designing All-Pay auctions*, investigating how the auctioneer’s utility is affected by the reward allocation. By simulating the behavior of learning participants, and predicting the outcomes of auctions using a neural network, our approach constructs a differentiable model for the auctioneer’s utility under various contest designs. Given the model, we then optimize the design by employing mirror ascent^12,13^, which allows optimizing the design while adhering to the fixed budget of the auctioneer.

*Our approach is flexible*: it can be applied to arbitrary mechanism design problems, including analytically intractable settings. It allows using various models for the behavior of participants. We apply Fictitious Play (FP)^14,15^ or independent reinforcement learning^16–18^.

We *empirically evaluate our framework on several contest design problems*. We study allocating a fixed reward budget in auctions with rank-order allocation of prizes, where the utility of a submission has diminishing returns in effort. We examine contests with few participants for which earlier research characterized the equilibrium behavior^19–21^.

We find that simulating participants’ behavior using Fictitious Play closely agrees with the equilibrium prediction. Note that FP is only known to converge to a Nash equilibrium in two-player zero-sum games^22^, and we examine All-Pay auctions, which are not zero-sum and have more than two participants. Nonetheless, we *empirically* show that FP does converge to the Nash equilibrium in the restricted settings where the Nash equilibrium is known. Furthermore, our framework identifies a design near the optimal design prescribed by the economic equilibrium analysis.

We then examine contests where the performance of a participant’s entry is determined by their exerted effort perturbed by random noise. Such uncertainty is a more realistic contest model, but the equilibrium behavior is unknown, highlighting the advantage of our approach. We show that designs with multiple prizes outperform awarding a single first prize in terms of auctioneer utility. As the variance of the random noise grows, we find that the optimal designs award larger second prizes, acting to protect bidders against the effect of the noise.

### Optimization goal and contest design space

We consider maximizing the auctioneer’s utility in a crowdsourcing contest (or the revenue of the auctioneer in an All-Pay auction). We examine contests that award multiple prizes based on the rank ordering of the performance of the participants. For instance, a contest may award a large first prize to the best performing contestant, and a smaller runner-up prize to the second best performer. Offering more prizes could incentivise more participants to exert effort, however a smaller top prize means that the maximum bid possible is also reduced.

Consider a contest with *n* bidders. The auctioneer decides on a division of a fixed total prize ${\bar{w}}$. The prize awarded to the $k{\text{th}}$ ranked player is denoted $w_k$, so $\sum _{k=1}^n w_k = {\bar{w}}$. We insist that prizes are decreasing with rank, i.e. that $w_1 \ge w_2 \ge ... \ge w_n$. Awarding a last-place prize reduces performance at equilibrium, as it reduces the incentive to exert more effort than other bidders, so we set $w_n = 0$. Bidders each choose a bid (effort level), with ${\mathbf{b}}$ denoting the vector of bids. Effort is costly, so the payoff for bidder *i* is the prize minus the effort:

1 $$\begin{aligned} s_i({\mathbf{b}}) = \sum _{j=1}^n w_j x_{i,j}({\mathbf{b}}) - b_i \end{aligned}$$

where $x_{i,j}({\mathbf{b}}) = 1$ when player *i*’s submission is ranked $j{\text{th}}$ in terms of its quality, and 0 otherwise.

In an all-pay auction, the auctioneer’s utility is a function of the winning bid. Hence, the efforts expended by the remaining losing bids is wasted. We can measure the inefficiency of an auction in terms of the expected wasted bids. In the setting where the auctioneer awards only the winning bidder and bidders play the Nash equilibrium, the expected maximum bid in an *n*-bidder auction is $\frac{n}{2n -1}$ and the expected bid is $\frac{1}{n}$^23^. Therefore, the expected inefficiency is *n* $\mathbb {E}$[bid] − $\mathbb {E}$[max bid] $= n (\frac{1}{n}) - \frac{n}{2n -1} = 1 - \frac{n}{2n -1}$.

Allocation is based on the ranking of the *realized* performance of the bidders. Some earlier work considers the realized performance to be deterministic given the bidder’s effort^19^, whereas others model the performance as a noisy, stochastic, function of the effort^24^. We also consider the performance $q_i$ as a noisy function of the effort $b_i$, indicating that participants have uncertainty about the exact effectiveness of their effort in producing high quality work. We model this uncertainty as random additive noise on the effort level: $q_i = \varepsilon _i + b_i$, where $\varepsilon _i$ is a random variable, drawn i.i.d for each contestant. We consider cases where $\varepsilon _i$’s distribution is either a zero-centered uniform or Beta distribution ($\alpha =\beta =\frac{1}{2}$) as well as the noiseless case (i.e. $\varepsilon _i=0$).

In this work, we assume a finite number of bid levels. For example, if bids are measured in a currency (e.g., dollars), there exists a minimal atomic amount (e.g., cents) and so the space of bids can be reasonably discretized. Similarly, if the bids are represented on a computer as floating point numbers, there also exists a minimal atomic amount given by floating point precision. We discuss the limitations of this assumption in the conclusion.

A bidding strategy $\sigma _i$ of participant *i* is a distribution over the bid levels. A set of bidding strategies $\sigma =(\sigma _1, \ldots , \sigma _n)$ is a *Nash equilibrium* if for any bidder *i* and any alternative strategy $\tilde{\sigma }_i$ (alternative distribution over bid levels) we have $s_i(\sigma ) \ge s_i(\tilde{\sigma }_i, \sigma _{-i})$, i.e. given the bidding strategy of others $\sigma _{-i}$, no player *i* wants to unilaterally deviate from their strategy $\sigma _i$ to any other strategy $\tilde{\sigma }_i$. It is only known how to derive Nash equilibria for specific All-Pay auction domains.

Given the realized performance of each contestant, the auctioneer receives a utility as a function *u* of the maximum performance, i.e. $u(\max _i q_i)$. The utility function *u* describes how the performance of the bidders translates into value to the auctioneer. We consider diminishing marginal returns on effort, modelled by a logarithmic utility function. Diminishing returns can also be used to model risk-aversion of the auctioneer. We model a fixed entry cost of *b* that does not contribute to the solution quality. For example, in the Netflix competition, contestants had to perform some work just to enter the contest, e.g. downloading data, efforts that provide no value to the auctioneer. Finally, we assume that the auctioneer has some existing default solution with a utility of 0. If no bid is better, the auctioneer uses the default solution and receives a utility of 0. Hence our auctioneer’s utility function is $u(q) = \max (\log (a(q - b)), 0)$, where *a* is a scale factor.

#### Goal

we seek the prize allocation $w=(w_1, \ldots , w_n)$ that maximizes the auctioneers’s expected utility $\mathbb {E}_\sigma (u(\max _i q_i))$ (given how participants would behave in the resulting contest). Multiple equilibria may exist in rank-allocation auctions. We focus on the *symmetric* case, where all bidders use the same strategy, a distribution over bids between 0 and the maximum prize available. In the noiseless case, theoretical analysis of the symmetric Nash is possible; for fewer than 5 bidders the density function of the symmetric equilibrium can be derived exactly, while for more bidders it can only be sampled from (see "Analytic results on noiseless auctions" section).

## Methods

**Figure 1:**
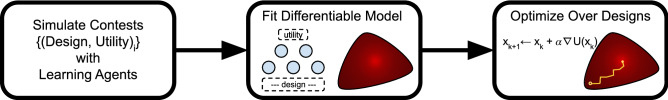
Diagram of the contest design process (Algorithm 2). Step (1) Simulate contests and agent learning to determine the utility of possible designs. Each data point represents a (contest design, auctioneer utility) pair. Step (2) Fit a deep network to predict auctioneer utility given contest design. Step (3) Optimize the output (utility) over the input (contest design) of the deep network to find the optimal design.

Our approach for automating the contest design process is illustrated in Fig. 1. Shortly, we simulate agent learning in contests under various designs and record the resulting auctioneer utilities. Next, we generalize from the training data by fitting a parameterized mapping from designs to utilities. As the mapping is differentiable, it allows gradient based optimization in the continuous space of designs, which we use to identify the optimal design under the model. We provide a detailed discussion of our method, given in Algorithm 2.

We begin by investigating a set $\mathscr {D}$ of possible contest designs. As discussed in "Optimization goal and contest design space" section, a design for *n* bidders is given by the reward distribution ${\mathbf{w}} = (w_1, \ldots , w_n)$, lying on the simplex (i.e. $\sum _{i=1}^n w_i = 1$ and each $w_i \ge 0$). Given a design $d \in \mathscr {D}$, our framework simulates how agents would learn to bid under this design. For the simulation, we use Fictitious Play^14^, one of the most prominent models for how an agent may learn and adapt their strategy; we also discuss other alternatives such as independent multi-agent reinforcement learning^16^. Our method is flexible and may use any model for agent learning in our simulation.

For a design $d \in \mathscr {D}$, the output of the simulation is the set of *bidding strategies* $\sigma _d$ of agents under this design, where $\sigma _d$ is a distribution over the bid levels. Given the bidding strategies $\sigma _d$ and contest simulation, we can also determine the expected utility $u_d$ for the auctioneer, as given in "Optimization goal and contest design space" section (the subscript *d* indicates the bidding strategies and the auctioneer’s utility depends on the contest design *d*).

By performing the simulation for many designs $d_1, \ldots , d_k$ chosen from the design space $\mathscr {D}$, we obtain a simulation dataset $\{ (d_i, u_{d_i}) \}_{i=1}^k$ where $d_i \in \mathscr {D}$ is a design and $u_{d_i}$ is the expected utility the simulation shows it would generate for the auctioneer (shown in the left of Fig. 1).

Using the simulation dataset, we train a differentiable model to predict the auctioneer’s utility $u_d$ under a contest design $d \in \mathscr {D}$ (including designs not observed during training). In other words, the true model for the auctioneer’s utility is a function $m : \mathscr {D} \rightarrow \mathscr {R}$, mapping any possible contest design in $\mathscr {D}$ to the utility it would provide to the auctioneer. We approximate *m* using a neural network, trained on simulation data, yielding the approximate function $m_{\theta } : \mathscr {D} \rightarrow \mathscr {R}$ ($\theta$ are model parameters). We use a simple feedforward network trained on many auction designs, depicted in the middle of Fig. 1.

Given $m_{\theta }$, we aim to identify designs resulting in high utility for the auctioneer; our goal is thus to “reverse engineer” the model, seeking inputs causing the model to output a high value reflecting high utility to the auctioneer. The model is differentiable, so we can calculate the gradient of the output with regard to the *inputs* $\nabla _{{\mathbf{w}}} m_{\theta }({\mathbf{w}})$, allowing gradient-based optimization.

A key challenge here is that the input design $(w_1, \ldots , w_n)$ must respect the auctioneer’s budget, i.e. $\sum _{i=1}^n w_i = {\bar{w}}$ and each $w_i \ge 0$. As illustrated on the right of Fig. 1, we perform the optimization while adhering to the auctioneer’s budget by employing a form of Entropic Mirror Ascent^12^, given in Algorithm 1 below. We now describe the data generation (Step 1) and design optimization (Step 3) in more detail.

### Data generation

We generate data to train the model $m_{\theta }$ by simulating the learning process of agents in auctions of a given design. The simulated auction receives bids as input and returns the rewards earned by the participants, as well as the auctioneer’s revenue. We use Fictitious Play (FP)^14^ as a model of agent learning. In FP, each agent adjusts a distribution over discrete bid levels by computing the best response to historical play.

We use FP as it is a well-established model of agent learning in strategic settings. However, there are alternative algorithms that can be used as the simulation method in our framework. Independent multi-agent RL (MARL) is a possible simulation alternative discussed in "Simulations using fictitious play and independent multi-agent reinforcement learning" section. See surveys for a detailed comparison of FP, MARL and other methods^25–27^.

### Design optimization

As discussed in "Optimization goal and contest design space" section, the design space is a convex set, the simplex: $\sum _{i=1}^n w_i = {\bar{w}}$ and each $w_i \ge 0$. In experiments, we let ${\bar{w}} = 1$ without loss of generality. Entropic Mirror Ascent^12^ is a non-euclidean gradient ascent method for convex optimization, designed for simplex constraints. The optimizer update rule for a design ${\mathbf{w}}$ is: ${\mathbf{w}} \leftarrow {\texttt{softmax}}(\log ({\mathbf{w}}) + \eta \nabla m_{\theta }({\mathbf{w}}))$ where $m_{\theta }({\mathbf{w}})$ represents the neural model’s predicted utility for input design ${\mathbf{w}}$. By inspection, ${\mathbf{w}}$ remains on the simplex after the update and $\log ({\mathbf{w}})$ is defined as long as ${\mathbf{w}} = {\mathbf{w}}_0$ is initialized to the interior of the simplex.

The simplex constraint for ${\mathbf{w}}=(w_1,\ldots ,w_n)$ is insufficient. Having prizes that are not monotonically decreasing in rank gives participants an incentive to attempt to obtain a lower rank (they get a higher prize for less effort). Hence, we want designs with strictly monotonically decreasing prizes and zero last prize (giving a prize to the lowest quality submission is wasteful, causing lower efforts). We propose a modified Entropic Mirror Ascent procedure to constrain iterates to this region of the simplex with a transformation.

For example, in a ($n$ $=$ $4$) four bidder contest, let ${\mathbf{w}}=[z_1+z_2+z_3, z_2+z_3, z_3, 0]$ where $z_i > 0$. $z_i$ denotes the marginal increase of the prize from that of rank $i-1$ to that of rank *i*. This sequence ${\mathbf{w}}$ is strictly monotonically decreasing. The simplex constraint implies $z_1 + 2z_2 + 3z_3 = 1$. Let *e* be the vector of coefficients, e.g., ${\mathbf{e}}=[1,2,3]$, and define $\tilde{z}_i = e_i z_i$. Then $\tilde{{\mathbf{z}}}$ lives on a simplex. We can run Entropic Mirror Ascent on $\tilde{{\mathbf{z}}}$ and transform back to ${\mathbf{z}}$ with ${\mathbf{z}} = \tilde{{\mathbf{z}}}/{\mathbf{e}}$. The update for $\tilde{{\mathbf{z}}}$ is

2 $$\begin{aligned} \tilde{{\mathbf{z}}}&\leftarrow {\texttt{softmax}}(\log (\tilde{{\mathbf{z}}}) + \eta \nabla _{\tilde{{\mathbf{z}}}} f(\tilde{{\mathbf{z}}})). \end{aligned}$$

We can rewrite this update in terms of ${\mathbf{z}}$ through a change of variables:

3 $$\begin{aligned} {\mathbf{z}}&\leftarrow {\texttt{softmax}}(\log (\tilde{{\mathbf{z}}}) + \eta J_{\tilde{{\mathbf{z}}}} ({\mathbf{z}}) {\nabla _{{\mathbf{z}}}} f(\tilde{{\mathbf{z}}})) \oslash {\mathbf{e}} \end{aligned}$$

4 $$\begin{aligned}{}&= {\texttt{softmax}}(\log ({\mathbf{e}} \odot {\mathbf{z}}) + \eta {\nabla _{{{\mathbf{z}}}}} f({\mathbf{z}}) \oslash e) \oslash {\mathbf{e}} \end{aligned}$$

where $J_{\tilde{{\mathbf{z}}}}({\mathbf{z}}) = {\texttt{diag}}({\mathbf{e}})^{-1}$ is the diagonal Jacobian matrix of derivatives of ${\mathbf{z}}$ w.r.t. $\tilde{{\mathbf{z}}}$, i.e., $J_{ij} = \frac{\partial z_i}{\partial \tilde{z}_j}$.

We formally express this idea in the transformation given in Algorithm 1 where $\odot$ and $\oslash$ denote element-wise multiplication and division respectively, $\Delta ^{n-1}_{int}$ denotes the interior of the simplex in $n-1$ dimensional ambient space, ${\mathbf{w}}[i$ $:$ $j] = [w_i, \ldots , w_{j-1}]$, ${\texttt{softmax}}({\mathbf{y}}) = \frac{e^{y_i}}{\sum _j e^{y_j}}$, ${\texttt{rev}}$ reverses an array, and ${\texttt{cumsum}}({\mathbf{y}})$ denotes the cumulative sum, i.e., $[y_1, y_1+y_2, \ldots , \sum _j y_j]$.
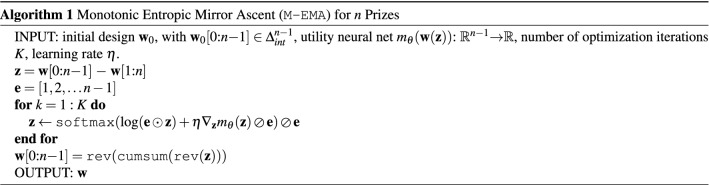


### Contest design using simulation, learning and optimization

Algorithm 2 is the overall auction design method, given informally in "Methods" section. It samples designs (we use a Dirichlet distribution $${Dir}_{n-1}(\alpha $$ $$=$$ $$1)$$), uses FP to simulate agent learning on each design, trains a neural network for predicting the auctioneers’s revenue and finally uses Algorithm 1 to optimize the design.
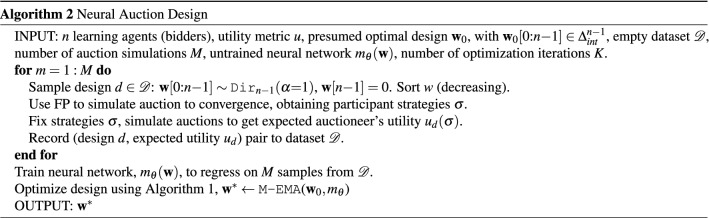


## Analytic results on noiseless auctions

We now very briefly discuss how one can solve for the closed form bidding strategies in crowdsourcing contests. A more detailed discussion of this can be found in contest theory textbooks^2^ and All-Pay auction papers^3–5,19,28–31^. Some prior work, such as^24^, has made progress analytically for specific noise models, but not for the models considered in this work.

We are interested in finding the symmetric Nash equilibrium for an All-Pay auction, as discussed in "Optimization goal and contest design space" section. In a symmetric Nash equilibrium, all bidders use the same bidding strategy $\sigma$, which is simply a distribution over the bid levels. In a symmetric Nash equilibrium, no bidder *i* wants to unilaterally deviate from $\sigma$ to an alternative bidding strategy $\tilde{\sigma }_i$. We write the CDF of a bidding strategy as *B*(*b*), and attempt to identify the symmetric Nash equilibrium.

First note that this equilibrium strategy is atomless. If it weren’t, agents bidding at the atom could achieve non-infinitesimal increases in their expected prize money by increasing their bid infinitesimally so as to outperform all other bids at the atom, therefore *B* would not be Nash. The expected prize money from bidding *b* when all bidders are following the bidding strategy *B* is given by:

$$\begin{aligned} \sum _{j=1}^n w_j G_j(B(b)) \text {, where }G_j(z) = {n-1 \atopwithdelims ()j-1}z^{n-j}(1-z)^{j-1} \end{aligned}$$

Each term of the sum is simply the value of the $j^{th}$ prize $w_j$ times the probability $G_j(B(b))$ that a bid of percentile *B*(*b*) achieves rank *j* against a set of $n-1$ independent bids drawn from *B*.

### Proposition

The symmetric equilibrium has expected value of 0 for participants.

### Proof

$B(0) = 0$ and *B* is continuous because *B* is atomless.

We write the expected utility when bidding *b* against opponents bidding according to *B* as *s*(*b*; *B*). Choose $\delta > 0$. The value *s*(*b*; *B*) of bids $b < B^{-1}(\delta )$ is bounded by the expected prize money under those bids, i.e. $s(b;B) \le \sum _{j=1}^n w_j G_j(B(b)) \le \sum _{j=1}^n w_j G_j(\delta )$.

Since $G_j(\delta )$ tends to 0 as $\delta$ tends to 0, for any $\varepsilon > 0$, $\exists \delta > 0$ s.t. bids $b \le B^{-1}(\delta )$ have an expected value $s(b;B) \le \varepsilon$. Furthermore, because $\delta > 0$, some such bids are in the support of *B*. Therefore there are bids in the Nash with value arbitrarily close to 0. Therefore no bid $\tilde{b}$ can have $s(\tilde{b};B) > 0$, since this would imply that there were bids that outperformed bids in the support of the Nash. A bid of 0 cannot win a prize, but also incurs no cost, so has a value of 0, so the value to bidders of the symmetric Nash must also be at least 0. $\square$

The proposition tells us that the symmetric Nash equilibrium *B*(*b*) satisfies:

5 $$\begin{aligned} s(b;B) = \sum _{j=1}^n w_j G_j(B(b)) - b = 0 \end{aligned}$$

This is a polynomial of order $n-1$ in *B*(*b*) for each value of *b*. Polynomials of up to order 4 can be solved analytically, therefore the CDF of the symmetric Nash can be expressed analytically for auctions with 5 or fewer bidders.

For any number of bidders, we can easily express the inverse-CDF using Eq. (5) as follows. We have $\sum _{j=1}^n w_j G_j(B(b)) - b$ so $b = \sum _{j=1}^n w_j G_j(B(b))$, and hence: $B^{-1}(y) = \sum _{j=1}^n w_j G_j(y)$ This allows sampling directly from the symmetric Nash equilibrium bid distribution in the noiseless setting, but relies on the fact that the probability of winning with a bid of *b* depends on *B* only through the value of *B*(*b*), which is not true in a noisy auction.

In the special case where the auction is noiseless and the auctioneer’s utility function $u: \mathbb {R}_+ \mapsto \mathbb {R}$ is strictly increasing, continuously differentiable and its inverse $u^{-1}$ is log-concave, Vojnović^2^ found that $\mathbb {E}[u(\max b_i)]$ under the symmetric Nash equilibrium is maximized by allocating the entire prize budget to the first prize.

Note however, that the inverse of $\log (a(x-b))$ is nowhere log-concave for $b>0$. Therefore the utility function considered in this work is not covered by this theorem. Indeed, we often found superior designs that awarded prizes to multiple places.

## Experiments

**Figure 2:**
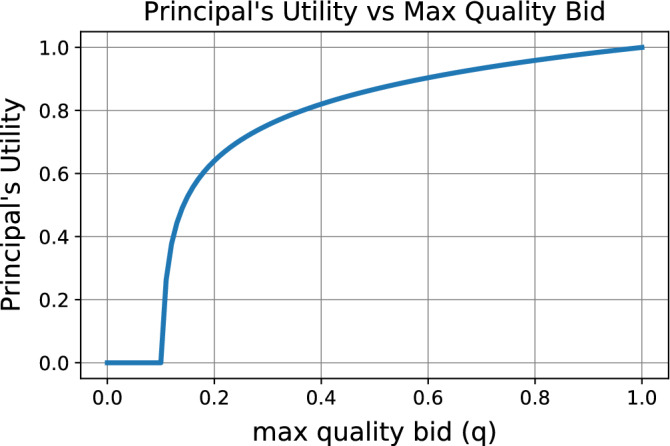
The auctioneer’s utility function: $\max (\log (a(q - b)), 0)$.

In "Optimization goal and contest design space" section describes assumptions one can make regarding the performance noise model and the utility of the auctioneer in crowdsourcing contests. We applied our proposed framework to optimize the design of crowdsourcing contests under various such assumptions. In all our experiments, we consider the auctioneer’s utility function to be the one given in "Optimization goal and contest design space" section, $u(q) = \max (\log (a(q - b)), 0)$, which reflects a risk averse auctioneer, with a minimal quality bar. We set $a=500$, $b=0.1$, and then rescale the utility to have a maximimum of 1 without loss of generality; see Fig. 2 for the shape of this utility.

In "Models with known equilibrium behaviour" section shows empirical results for a domain with three or four bidders, and noiseless performance. As shown in "Optimization goal and contest design space" section, in this model the symmetric equilibrium strategy is *known*. Our analysis shows that the FP simulation results in agent behavior that is extremely close to the Nash equilibrium prediction. After fitting a differentiable model and optimizing the design using Algorithm 2 we obtain the same optimal design as the equilibrium based analysis.

In "Models with unknown equilibrium behaviour" section considers settings where the equilibrium behavior is *not known*, so standard economic techniques struggle to recommend an optimal design. We consider 10 participants and various performance noise models, and apply our framework to identify the optimal design. We show that our designs award money to a few top entrants. As the variance of performance noise increases, optimal designs award more prizes, and larger prizes to the runner-up in the contest.

### Method details

We ran FP for 100, 000 iterations with a discretization of 1001 effort levels for the bid interval [0, 1]. We are searching for a symmetric equilibrium so all bidders played using the same bid distributions, i.e. using Fictitious Self-Play.

For our neural network $m_{\theta }$, we have used a simple feedforward network with 2 hidden layers, 256 neurons per layer, and ReLU nonlinearities. We trained the network for 10, 000 iterations using the Adam optimizer^32^ with a learning rate of $10^{-3}$ and default hyperparameters $\beta _1=0.9$, $\beta _2=0.999$. We optimize using mini-batches of size 50 for the three and four bidder auctions and 1, 000 for the 10 bidder auction.

We initialized designs such that the first prize was given 0.9 and all remaining marginals were given a constant $z_{i>1} = c$ such that the prizes sum to 1. We performed 100, 000 iterations of E-EMA with a learning rate of 0.1 for the 3 bidder auction and 200, 000 iterations with a learning rate of 0.001 for the 10 bidder auctions.

All experiments were written in Python+numpy^33^ and run on a single CPU selected from a heterogeneous cluster of CPUs. An Intel(R) Xeon(R) W-2135 CPU @ 3.70GHz was representative CPU (6-core).

### Models with known equilibrium behaviour

Consider a setting with three or four bidders, and with no performance noise. The first step in our framework is simulating agents who learn from repeated interaction in the contest, by applying FP. We first investigate whether the predictions of FP agree with the Nash equilibrium strategies. In general FP *may not* converge to a Nash equilibrium as an All-Pay auction is *not* a constant-sum or dominance solveable game^34,35^. Furthermore, the solution found with Fictitious Play is to a discrete version of the auction (where bids take one of a discrete set of values), whereas the analytic solution is for the case where bids can take any real value.

**Figure 3:**
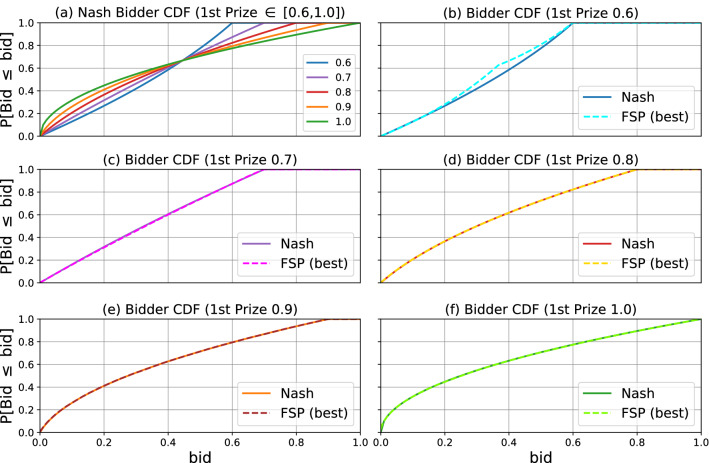
Bidding strategy CDFs. (**a**) shows the CDFs for each of five different contest designs assuming the bidder plays the Nash distribution. The first prize is listed in the legend; the second prize equals one minus the first; no prize is given to the third bidder. The remaining plots (**b**–**f**) compare the Nash CDF with the CDF learned by Fictitious Self-Play for different first prize amounts.

Figure 3a shows the symmetric Nash equilibrium bidding strategy, as the cummulative distribution function (CDF) of the distribution over bid levels, under multiple three bidder contest designs, characterized by the prize for the top rank (the remainder prize goes to the second rank, and the prize for the third rank is zero). The remaining plots of Fig. 3 each examine one design (characterized by the first rank prize), and plot the bid CDFs of the Nash equilibrium versus those outputted by FP. Figure 3 shows that the FP output closely matches the Nash equilibrium.

We estimate inefficiency of the auction empirically for the approximate equilibrium strategy returned by FP for using 10, 000 Monte-Carlo simulations for the 3-bidder auction with varying prize structures and report them in Table 1.

**Table 1:** Inefficiency of Noiseless 3-Bidder Auctions.

First prize	Inefficiency
0.6	0.520
0.7	0.491
0.8	0.460
0.9	0.430
1.0	0.399

Table 1 confirms that the approximate equilibrium strategy returned by FP in Fig. 3 (f) closely matches the first prize only auction inefficiency of 0.4 as predicted by the formula discussed in "Optimization goal and contest design space" section: $1 - \frac{3}{6-1} = \frac{2}{5} = 0.4$. Recall that giving all prize money to the max bid maximizes auctioneer revenue in the noiseless setting. So it is interesting to note from this table that a reduction in inefficiency correlates with an increase in auctioneer revenue.

**Figure 4:**
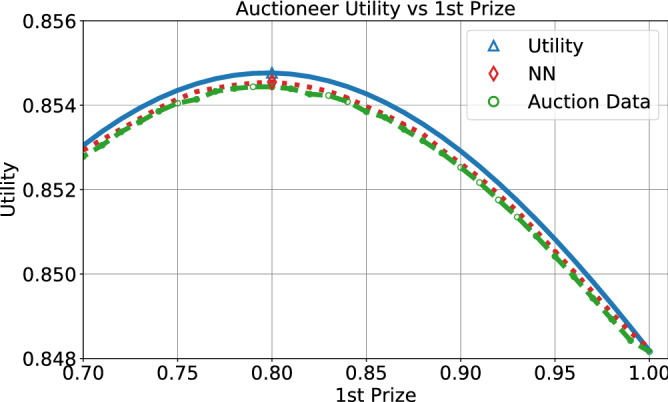
Auctioneer utility as a function of the first rank prize, given by the analytical Nash CDFs, the simulation data, and the trained network. Markers denote maxima.

The next phase in our pipeline takes the dataset of simulation outcomes under various designs, and trains a neural network to predict the auctioneer’s utility in any given contest design (attempting to generalize to unsimulated designs). Figure 4 compares the auctioneer’s utility under Nash bidding against the prediction of our trained model for various designs (characterized by the first rank reward, shown on the x-axis). We observe that the simulation results for the auctioneer’s utility are consistently very slightly below the Nash-based analytical solution. The model has an almost perfect fit to the simulation results.

The final step of our method is optimizing the contest design given the model (Algorithm 1). The optimal design is marked in Fig. 4, for both the Nash-based curve and our method. These match almost perfectly (the location on the x-axis is almost identical), indicating our method finds the same optimal design as prescribed by the Nash equilibrium analysis.

**Figure 5:**
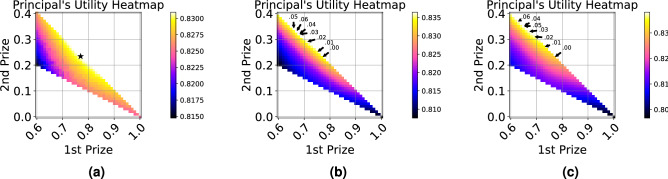
(**a**) Heatmap for the auctioneer’s utility for various four bidder contest designs. The star marks the optimal design. (**b**) Heatmap with realized bids drawn from an interval [bid$-d,$bid$+d]$ uniformly. Plotted on top of the heatmap are arrows to the optimal designs for different values of *d* annotated on the map. (**c**) Heatmap with realized bids drawn from an interval [bid$-d,$ bid$+d]$ according to Beta($\frac{1}{2},\frac{1}{2}$), $d=0.06$.

Finally, we explore a four bidder contest to investigate the effect of possible designs on the auctioneer’s utility. Figure 5a shows a heatmap for the auctioneer’s utility for possible designs. The x-axis is the reward $w_1$, the prize for the first rank, and the y-axis is the reward $w_2$ for the second rank (the lowest rank gets no reward $w_4=0$, and the third rank reward is $w_3 = w - w_1 - w_2$). Figure 5a shows that the utility is fairly robust to designs with a high first prize, i.e., $w_1 \in [0.7-0.9]$ and third prizes $w_3 < 0.1$. However, good designs with a low first prize (e.g. $w_1 < 0.7$) offer no reward to the third rank. This indicates that in settings with many participants we might expect a greater distribution of reward across top prizes, but the auctioneer’s utility may be fairly robust around the optimal design.

We also empirically estimate the exploitability of the strategy FP learns in the 4-bidder setting under the predicted optimal auction design (see Fig. 5a, ${\mathbf{w}}^*=[0.77, 0.23, 0.00]$). Exploitability of a strategy set $\sigma$ is measured as the maximum expected amount a single bidder can gain by deviating to another bidding strategy. To measure exploitability, we first simulate the auction where all bidders deploy the learned FP strategy and record the average bidder payoff. We run 10, 000 Monte-Carlo simulations to estimate this value. We then consider every possible deviation to a pure bidding strategy a bidder can make. As before, we consider 1, 001 bid levels. For each of the 1, 001 bid levels, we let one bidder play that pure bid strategy while the remaining play the learned FP strategy. We then calculate the gain a player can expect by deviating to one of these pure bid strategies by subtracting off the expected payoff of the learned FP strategy. We estimate the exploitability to be 0.0003, which means a bidder can expect to gain at most 0.0003 if they attempt to deviate.

### Models with unknown equilibrium behaviour

We explore contests where each participant’s bid is perturbed by random noise to yield their performance. We consider noise following either a uniform or a Beta($\frac{1}{2},\frac{1}{2}$) distribution. Due to the noise distribution, the Nash equilibrium bidding strategy is not known for this setting. We apply our method on such contests, and investigate how the optimal design is affected by the noise distribution.

Figures 5b and c show heatmaps of the auctioneer’s utility in the noisy setting (uniform noise on the left and Beta noise on the right), under different contest designs. Similarly to Fig. 5a the axes are $w_1$ and $w_2$, the last prize is $w_4=0$, and $w_3 = 1 - w_1 - w_2$. Figure 5b and c show that as more noise is introduced to the bids, the optimal designs tighten around more evenly distributing reward across the top two bids (in both cases, $w_3=0$ in the optimal design). In other words, as the noise increases, the optimal design transfers more reward from the top rank to the one below it. An exploitability analysis suggests bidders can expect to gain at most 0.02 if they choose to deviate from the learned FP strategy under the predicted optimal auction design for uniform noise with $d = 0.06$ (see Fig. 5b, ${\mathbf{w}}^*=[0.67, 0.33, 0.00]$).

**Figure 6:**
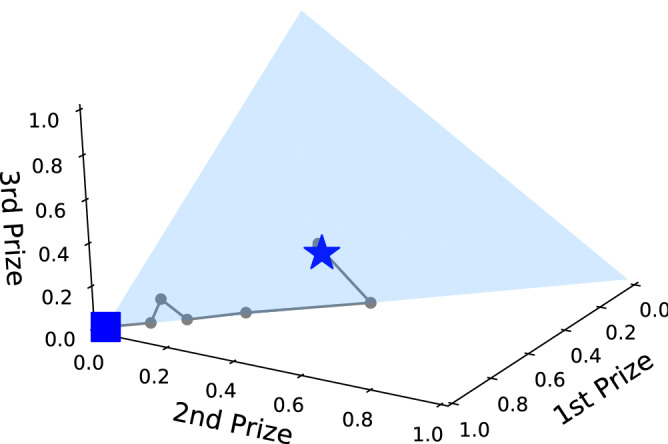
First three prizes of the optimized ten bidder contest plotted on top of a simplex for reference. Each point denotes the optimal design for a different noise level. The square marks zero noise with the trajectory ending at the star with realized bids drawn uniformly from bid $\pm 0.06$.

We now investigate contests with more participants, showing how performance noise affects the optimal design. Figure 6 shows the optimal design for $n=10$ participants, for different performance noise levels. We only illustrate the top 3 prizes in a 3D plot (lower ranks typically get very little or no reward in the optimal design). Figure 6 shows that increasing the noise makes the optimal design spread the reward more evenly among the top ranks. Table 2 shows this as a table, giving the optimal design and inequality in prize levels. An exploitability analysis suggests bidders can expect to gain at most 0.002 and 0.09 if they choose to deviate from the learned FP strategy in the noiseless and uniform noise setting ($d = 0.06$) respectively under the predicted optimal auction design (see first and last rows of Table 2 for optimal designs). Note that in the noisy setting, realized bids are drawn from an interval of size 0.12 ($2\times d$). Therefore, we consider 0.09 to still be a relatively low level of exploitability for the noisy 10-bidder auction.

**Table 2:** First three prizes of the optimal ten bidder auction of Algorithm 2.

*w*	$w^*_1$	$w^*_2$	$w^*_3$	Gini
0.000	1.000	0.000	0.000	0.900
0.002	0.916	0.084	0.000	0.883
0.004	0.850	0.075	0.075	0.855
0.006	0.848	0.152	0.000	0.870
0.008	0.733	0.260	0.004	0.844
0.010	0.500	0.500	0.000	0.800
0.020	0.500	0.500	0.000	0.800
0.030	0.500	0.500	0.000	0.800
0.040	0.471	0.325	0.204	0.753
0.050	0.461	0.319	0.209	0.746
0.060	0.443	0.319	0.152	0.724

Prizes of fifth rank and higher are all zero. Width of the uniform distribution around bidder bids given in column *d*; Gini index of the design is in the last column.

Finally, we investigate the limitations of our approach. Our framework may suggest a sub-optimal design due to multiple issues. First, the simulation of how participants learn may not be an accurate model of their behavior. Second, the differentiable model learned for predicting the auctioneer’s utility may an inaccurate approximation of the true function (i.e. we may have neural network generalization error). Third, the optimization procedure (Algorithm 1) may converge on a local rather than global optimum.

**Figure 7:**
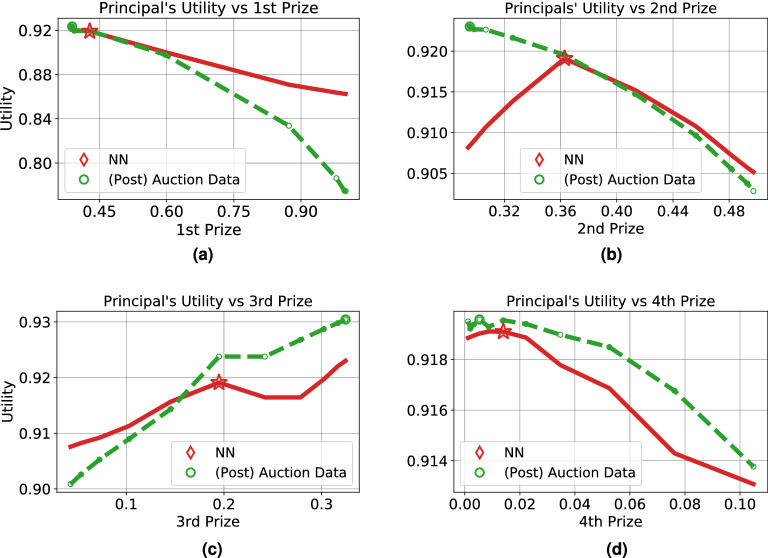
Utility for ten bidder contest vs the top four prizes with realized bids drawn uniformly from bid $\pm .06$. Plots shown on different scales and slices of the design space.

Figure 7 illustrates the generalization error contrasting the auctioneer’s utility when running the FP simulation and when predicting it using the trained model on previously unobserved designs. We note that the neural network’s predictions are slightly different from the simulation data, though they follow similar trends. Further, Fig. 7 also marks the location of the optimized design suggested by Algorithm 2 with a star, showing errors may occur due to convergence to a local rather than global optimum (e.g., Fig. 7c).

As discussed above, the variability in the final design output by the neural network can attributed to both the training data and the local learning rule (gradient ascent) that we use to search for the optimal design. Our local learning rule is not immune to local optima and so it may return a different output on each run. In an effort to quantify that variability, we repeat the search for an optimal design using varying proportions of the training data. For each training set size, we measure the average pairwise Jensen-Shannon distance between the optimal designs generated from 10 differnt trials. We focus on the 10-bidder auction with uniform noise ($d = 0.06$). In Table 3, we find that the variability in the output does indeed drop as the size of the training dataset increases.

**Table 3:** Variability in auction design predicted by neural network vs training set size.

Proportion of training data used (%)	Average pairwise Jensen-Shannon distance between designs
10	0.329
50	0.212
75	0.105
100	0.081

## Simulations using fictitious play and independent multi-agent reinforcement learning

Our simulation phase used Fictitious Play (FP)^14,15^. An alternative is using independent multi-agent reinforcement learning^16–18^. We provide empirical evidence showing that FP better matches equilibrium based analysis.

**Figure 8:**
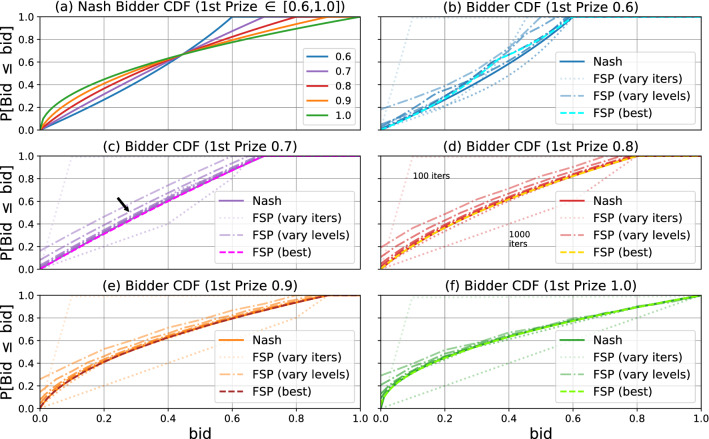
Bidder CDF comparison. (**a**) shows the CDFs for each of five different auction designs assuming the bidder plays the Nash distribution. The first prize is listed in the legend; the second prize equals one minus the first; no prize is given to the third bidder. The remaining plots compare the Nash CDF with the CDF learned by Fictitious Self-Play for different first prize amounts. (**b**)–(**f**) Show the effect of training iterations and discretization granularity on the final CDF. The arrow in (**c**) highlights a common trend where the CDF converges to Nash from above as a finer discretization is introduced: coarser discretizations lead to under bidding and, in turn, underestimates of the auctioneer utility. (**d**) Shows that increasing training iterations reduces error, but in a less structured manner than bid granularity.

In FP each agent assumes the opponents play a stationary (mixed) strategy, and in each round every player chooses the best response to the empirical frequency of play of their opponents. Figure 8 investigates the impact of the number of rounds on the learned bidding strategies (distribution over bid levels), contrasting it with the Nash equilibrium for the game. It shows the same results as Fig. 3 but for varying number of FP rounds and discretization granularities for bid levels.

Figure 8 indicates that the number of FP rounds and the granularity of discretization of bid levels have an impact on the learned bidding strategies. However, the results are somewhat robust to the choice of these parameters, yielding similar bidding strategies under many settings.

We examine independent multi-agent reinforcement learning (MARL) for the simulation phase. MARL methods have recently become popular for modeling agent behavior in multi-agent environments. The *n*-bidder All-Pay auction can be formulated as a multi-agent reinforcement learning problem as follows. The environment contains only a single state *s*, where every episode begins. Each bidder *i* then simultaneously makes a bid $b_i$. Finally, the environment calculates and distributes rewards to the agents according to the payoff function in Eq. (1). Formally, this is a one state Markov game^16^, i.e., a multi-agent bandit, with the relevant details given in Table 4.

**Table 4:** All-pay auction as a multi-agent reinforcement learning problem.

State-space (*S*)	one “pre-bid” state, i.e., $S = \{s\}$ is a set with a single state
Bidder *i* Action-space ($B_i$)	$B_i = \{0, \ldots , 1\}$ bid effort levels, let $b_i$ be the effort level selected by player *i*
Joint action-space (*B*)	$B = B_1 \times \ldots \times B_n$, let ${\mathbf{b}}$ be the vector of all players’ selected bid levels
Bidder *i* Reward	$R_i(s, b) = s_i(b)$ as defined in Eq. (1).

**Figure 9:**
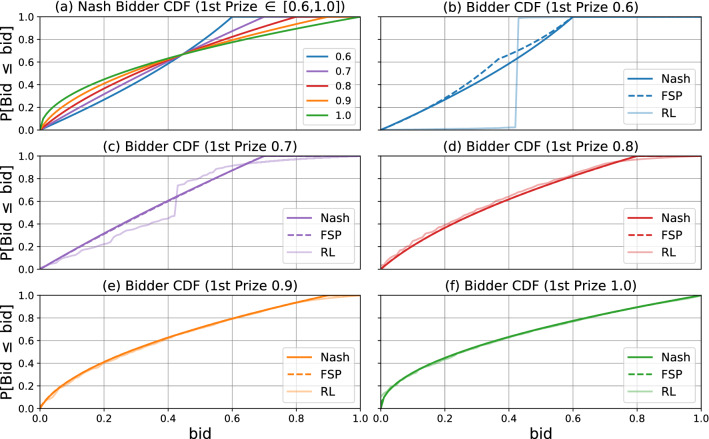
Bidder CDFs with reinforcement learning simulation. (**a**) shows the CDFs for each of five different auction designs assuming the bidder plays the Nash distribution. The first prize is listed in the legend; the second prize equals one minus the first; no prize is given to the third bidder. The remaining plots (**b**–**f**) compare the Nash CDF with the CDFs learned by Fictitious Self-Play and REINFORCE respectively using their optimal hyperparameter configurations (iterations, bid levels, learning rate, batch size). Both REINFORCE and Fictitious Self-Play agree closely with the Nash equilibrium when the first prize $\ge 0.8$. For smaller prizes, there is a larger discrepancy. FP is consistently closer to the equilibrium than REINFORCE.

We use independent REINFORCE^36^, and investigate the bidding strategies learned by the agents. Bidding strategies learned using MARL are shown in Fig. 9, contrasted with the equilibrium and strategies learned via FP (similarly to Figs. 3 and  8). For large first prizes (0.8 or higher), all methods yield a similar distribution. However, there is a deviation for lower top prizes, where the RL distribution has a step function curve.

Figure 9 indicates that the model for agent learning may impact the assumed bidding strategies (and hence on the choice of a design). Ultimately, we feel like this is a modelling decision on the user’s side: in order to choose a good design, one must first determine what is a reasonable model of the learning behavior of agents.

For All-Pay auctions, using FP yields results that are more similar to those of traditional Nash equilibrium analysis. In contrast, if one believes agents are more likely to be reinforcement learners, an alternative bidding strategy is a likely outcome. One possible choice is a conservative approach, where one only considers designs where there is a consensus between simulation learning rules (e.g. FP or MARL). In this case, one may opt for a large top prize, as in this case, the different models for agent learning behaviour agree with each other.

## Related work

All-Pay auctions have received significant attention in the economic literature, including recently published surveys and books focusing on the topic^2,37^. We propose a neural approach to designing crowdsourcing contests.

Earlier work has carried out equilibrium analysis for restricted models of All-Pay auctions^3,4,19,24,28,29^, including the impact of risk-aversion^7,38^. Such an equilibrium analysis can potentially be viewed as a model of how “rational” participants may bid in such settings, reflecting specific assumptions on how people are likely to engage in strategic situations.

However, the actual behavior of human participants may significantly deviate from the predicted equilibrium in many games or strategic settings^39,40^. Empirical evaluation of how people actually bid in such settings has revealed discrepancies with the predictions of the equilibrium based analysis^9,41,42^. Such empirical work suggests that people employ simple learning heuristics^37,41^. In particular, the best-response heuristic has been analyzed in the auction setting^43^. In fact, other work has defined alternative equilibrium behaviour in terms of the stationary distribution of evolutionary learning dynamics observed in nature^44^. Given this evidence, we focus on a learning-based model of bidding behavior.

We examine an auctioneer deciding on a rank reward allocation, in order to maximize its utility. This broadly falls within the field of *mechanism design* or *auction design*^45–47^, a subfield in economics, seeking to decide the “rules of the game” so as to achieve desired outcomes.

Typically, auctions are designed *manually* by economists seeking to maximize revenue^45,48,49^. In contrast, we *automate* the process, similarly to work on *automated mechanism design*^50–58^. In other words, we design a process that allows machines to take on the burden of the work of analyzing potential rules of an auction or a game and selecting ones that are likely to lead to desired outcomes. In contrast to much of the work in the space of automated mechanism design, which deal with first-price and second price auctions^59^ or extensions such as Vickrey-Clarke-Groves mechanisms^60,61^ we focus on All-Pay auctions, where all participants have an identical value for the prize.

We use machine learning to search the design space, akin to recent deep-learning mechanism design frameworks for other auction or mechanism types^62–70^. Much of this earlier work considers a family of auction rules for which one can analytically compute the equilibrium behavior of agents (in some cases a dominant strategy equilibrium, and in others refinements of a Nash equilibrium); when the equilibrium behavior is known, it can serve as a model of how participants are likely to behave under a design of an auction. We consider domains where the equilibrium of the game is unknown, and must thus employ other means to predict the behavior of participants. Hence, in contrast to the above work, we leverage agent learning of the auction^71,72^. Learning agents are increasingly capable of solving complex problems; using such capable agents for mechanism design holds the promise of optimizing the design of mechanisms in more complex settings than previously possible.

## Conclusion

Our empirical analysis shows the promise of automated mechanism design based on deep learning. However, our technique has several limitations, such as the dependence on a good model for the learning of agents, and errors introduced by inaccurate function approximation and converging on local optima.

Broadly, our technique is a form of automated mechanism design^51,73^ that combines deep learning and multi-agent simulation. We hope that these results would trigger further research on using neural networks to design mechanisms. For example one could identify mechanisms that are more robust to false-name attacks^74–78^ or collusion^23,79–84^. While we have focused on all-pay auctions, but we believe similar techniques could be used in broader settings, such as pricing crowdsourcing markets, effort prediction^85^, or principal-agent settings^86^.

Several questions are open for future research. We assume a finite number of bid levels. If the discretization used by bidders is heterogeneous, a coarse discretization could leave one bidder vulnerable if other bidders are using a more fine-grained discretization. For example, a bidder bidding in cents could undercut a bidder bidding in dollar increments only. In order to approximately counter such arbitrage, bidders may want to randomize over two adjacent bid levels (coarse discretization) to effectively achieve bidding in between two levels (finer discretization). How can we best model the continuous setting and how can be design auctions for settings where bidders might be using different discretization levels?

In addition, can our methods generalize well to other mechanism design domains such as other types of auctions? What are good models of agent learning in other strategic settings? Do such models do a good job in characterizing the bidding behavior of *human* participants? Finally, can better methods be devised to optimize over designs?

## Data Availability

All data and information necessary for replicating these experiments is contained in the manuscript. No additional external datasets were used.
